# Evaluation of Selected Folk Herbs on the Fertility of Sprague Dawley Male Rats: Biochemical, Histological, and Molecular Investigations

**DOI:** 10.3390/life14121620

**Published:** 2024-12-06

**Authors:** Rana R. Khalaf, Salma Khazaal, Noura S. Abouzeinab, Mahmoud I. Khalil

**Affiliations:** 1 Department of Biological Sciences, Faculty of Science, Beirut Arab University, P.O. Box 115020 Riad El Solh, Beirut 11072809, Lebanon; rrk297@student.bau.edu.lb (R.R.K.); or mahmoud_ibrahim@alexu.edu.eg (M.I.K.); 2 Department of Nutrition and Dietetics, Faculty of Health Sciences, Beirut Arab University, P.O. Box 115020 Riad El Solh, Beirut 11072809, Lebanon; s.khazaal@bau.edu.lb; 3 Molecular Biology Unit, Department of Zoology, Faculty of Science, Alexandria University, Alexandria 21568, Egypt

**Keywords:** fertility, spermatogenesis, histology, testosterone, Western blotting, herbal medicine

## Abstract

Scientists have shown great interest in traditional plant extracts, particularly *Lepidium sativum* (LS), *Origanum majorana* (OM), *Ferula hermonis* (FH), and *Eruca sativa* (ES), which are frequ ently used to improve health. Recently, attention has been directed toward their influence on spermatogenesis and male fertility. Hence, the objective of this study was to explore their impact on male rats’ fertility. Antioxidant activity and total phenolic content (TPC) were determined, along with the identification and quantification of phenolic compounds. Oral administration of aqueous extracts was performed individually or as a mixture (MIX) at a dose of 100 mg/kg in 28 male Sprague Dawley rats over a 60-day period. Organ weight, sex hormone concentrations, sperm parameters, oxidative stress markers, histological and morphometric analysis, and protein expression levels were investigated. OM and MIX showed the highest TPC and antioxidant activities, and MIX possessed the highest polyphenolic constituents. Elevated serum testosterone, epididymal sperm concentration, testes glutathione levels, and histomorphometric parameters were manifested in all groups, especially in MIX. MIX group also displayed elevated levels of vimentin, protein kinase B, and mTOR expression in the testes, complemented by declined expression of Phosphatase and Tensin Homolog (PTEN). In conclusion, these findings propose that these extracts, especially MIX followed by OM, enhance fertility by stimulating spermatogenesis.

## 1. Introduction

Infertility is described by the inability to attain pregnancy after committing to consistent unprotected sexual activity for a period of 12 months or longer [1]. Generally, about 20% of cases of infertility are caused by male-related factors that comprise male tract infection, varicocele, and hormonal dysfunction and can occasionally remain unidentified [2]. The principal diagnostic indicator of infertility and subfertility in men is characteristically an insufficiency in semen quality, marked by low sperm count, abnormal morphology, and deficient motility [3]. Millions of sperm are produced each day during spermatogenesis, which occurs in the seminiferous tubules (STs) and is directly related to male fertility.

Spermatogenesis is tightly controlled by complex hormonal signaling pathways [4]. The primary androgen involved in this process is testosterone, which is produced by Leydig cells and serves three important functions: maintaining the blood–testes barrier, promoting Sertoli–spermatid adhesion, and facilitating the release of mature sperm. Moreover, it is involved in the development of secondary male sexual characteristics, and together with follicle-stimulating hormone (FSH), it supports spermatogenesis, while luteinizing hormone (LH) controls its production [5]. A delicate balance between renewal, differentiation, and commitment of spermatocytes to meiosis is obligatory in spermatogonia stem cells (SSCs) for spermatogenesis. Abnormal expression of genes or transduction of signals, mostly in the testes, are the fundamental causes of spermatogenic malfunctions and infertility in males. The PI3K/AKT/mTOR signaling pathway plays an important role in several key processes of male reproduction. It regulates the self-renewal and differentiation of spermatogonial stem cells, the proliferation and maturation of spermatogonia, in addition to contributing to the function of testicular somatic cells and the regulation of the hypothalamic–pituitary–gonadal (HPG) axis [6]. PTEN (phosphatase and tensin homologue deleted on chromosome ten) is a down regulator of the PI3K/AKT pathway [7]. Cytoplasmic intermediate filaments are considered an important constituent of the cytoskeleton of Sertoli cells. The primary component of these intermediate filaments is vimentin [8].

The treatment of male infertility may possibly include medication, surgical intervention, and assistive reproductive technologies, namely in vitro fertilization [9]. As a less invasive possibility, alternative therapies, including herbal plants, are accessible [10]. Several studies have revealed the promising effects of countless botanical medicines on sperm parameters and overall male fertility [11]. Among these plants, *Lepidium sativum* (LS), *Origanum majorana* (OM), *Ferula hermonis* (FH), and *Eruca sativa* (ES) are all native to the Mediterranean region. LS seed suspension was found to treat hyperprolactinemia and decrease the risk of infertility in male mice [12]. OM oil extract revealed a high ability to prevent male fertility and infertility disorders, particularly in overweight rat models [13,14]. The traditional uses of dried FH roots comprise the management of frigidity and impotence, as well as the improvement of sexual desire [15]. However, Homady et al. [16] proposed that FH root extract has toxic effects on female and male fertility in mice. The testosterone levels and fertility potential of male rats were ameliorated by the ethanolic extract of ES leaves [17]. Vitamin E (α-tocopherol), a critical antioxidant required for the maintenance of mammalian spermatogenesis, is a potent antioxidant that dwells in the cell membrane and is essential to protect cells by breaking the oxidative chains [18]. Sertoli cells and pachytene spermatocytes are the two tissues in which vitamin E is most abundant. Due to the oxidative stress in the testes instigated by vitamin deficiency, spermatogenesis can be altered [19].

Several plants commonly found in local markets and used in traditional medicine have been extensively documented and studied, providing scientific evidence to support their traditional applications in human health. However, the possible effect of consuming an aqueous combination including the indicated folk herbs (LS, OM, FH, and ES) on the reproductive system of male Sprague Dawley (SD) rats remains unknown. Consequently, the present investigation’s purpose was to explore the separate and combined effects of the aqueous extracts of LS, OM, FH, and ES on male SD rats’ fertility after 60 days of daily oral administration, with vitamin E being considered as a positive control.

## 2. Materials and Methods

First, the total phenolic content (TPC) was determined, along with the polyphenols present and the antioxidant activity in the selected herbs and their mixture. Then, fertility was investigated by examining histopathological and morphometric measurements, performing hormonal and molecular evaluations, and evaluating oxidative stress markers. Male SD rats were chosen as a comprehensive animal model that exhibit a great resemblance to human developmental and physiological processes, despite certain differences, which offers significant clinical and research relevance.

### 2.1. Chemicals

The subsequent chemicals were acquired from Sigma Aldrich (Steinheim, Germany): gallic acid (3,4,5-trihydroxybenzoic acid), sodium carbonate (Na_2_CO_3_), Trolox (6-hydroxy-2,5,7,8-tetramethylchro mane-2-carboxylic acid), Folin–Ciocalteu reagent, and DPPH (2,2-diphenyl-picrylhydrazyl), along with HPLC standards (protocatechuic acid, gallic acid (GA), hydroxybenzoic acid, chlorogenic acid, catechin, caffeic acid, rutin, *p*-coumaric acid, ellagic acid, and trans-cinnamic acid). Protease inhibitor III, NBT (nitro blue tetrazolium), GSH (reduced glutathione), and DTNB (5,5′-Dithiobis 2-nitrobenzoic acid) were obtained from Sigma-Aldrich (St. Louis, MO, USA).

### 2.2. Preparation of Plant Extracts

The roots of *Ferula hermonis,* dried *Origanum majorana* leaves, *Lepidium sativum* seeds, and aerial parts of *Eruca sativa* were purchased from different local markets, stored in the dark, and identified in the botanical herbal garden for Lebanese medicinal and aromatic plants at Beirut Arab University (BAU) Research Center for Environment and Development (RCED)—Bekaa Campus, Lebanon (https://www.bau.edu.lb/Research-Center-for-Environment-and-Development/Projects-BAU-Herbal-Garden-for-Lebanese-Medicinal-and-Aromatic-Plants, accessed on 10 September 2022). The selected plant parts were shade-dried, sliced, and ground, and the water extracts were prepared by combining 25 g of each plant with 500 mL of distilled water. After 30 min of heating in a water bath at 95 °C, the extract was vacuum filtered by Buchner funnel filtration. The acquired solution was distributed into aliquots and stored at −4 °C until use. Vitamin E was purchased from Webber Naturals^®^, Coquitlam, BC, Canada.

### 2.3. Total Phenolic Content (TPC)

The Folin–Ciocalteu method was used to assess TPC as previously described by Singleton et al. [20]. In summary, 200 µL of plant extract was mixed with 800 µL of Na_2_CO_3_ 7.5% (*w*/*v*) and 1000 µL of Folin–Ciocalteu reagent (diluted 1/10 *v*/*v*). The solution was left to incubate at 60 °C for 10 min and then at 4 °C for another 10 min. Absorbance measurements were performed using a UV-Vis spectrophotometer (GENESYS 10 UV, Thermo Electron Corporation, Waltham, MA, USA) at 750 nm. With gallic acid serving as a standard, a calibration curve was used to determine the TPC, which was expressed as milligrams of gallic acid equivalents (GAE) per gram (mg GAE/g).

### 2.4. DiPhenyl-2-PicrylHydrazyl Free Radical Scavenging Activity (DPPH) Assay

The aptitude of phenolic compounds to reduce DPPH radicals was employed to investigate their free radical scavenging activity [21]. Briefly, 1.45 mL of DPPH (0.06 mM) was mixed with 50 µL of the plant extract, Trolox (positive control), or methanol (negative control). Following an incubation period of 30 min at room temperature in the absence of light, measurements of the absorbance (Abs) were performed at 515 nm. The blank used was pure methanol, and Trolox was used as the standard to generate a calibration curve. Finally, the antioxidant activity of the samples was obtained utilizing the following formula:

Antioxidant activity (%) = (Abs (negative control) − Abs (sample)/Abs (negative control)) × 100(1)

### 2.5. Identification and Quantification of Polyphenols by High-Performance Liquid Chromatography (HPLC)

The HPLC analysis was performed for plant extracts’ phenolic compounds’ identification and quantification. The apparatus for HPLC analysis was an Agilent 1100 Series system combined with a Zorbax column oven, an autosampler, and a diode array sensor (Barcelona, Spain). A C18 column (250 × 4.6 mm; 5 μm) (Thermofisher, Waltham, MA, USA) was considered for the separation of phenolic compounds. Furthermore, the standards of rutin, catechin, hydroxybenzoic acid, protocatechuic acid, ellagic acid, chlorogenic acid, *p*-coumaric acid, and gallic acid were used to recognize and determine the compounds present in the extracts. The mobile phase consisted of acidified nano-pure water at pH 2.3 with HCl (A) and methanol (B) of HPLC grade. The elution procedure was performed under isocratic conditions, starting with 85% A and 15% B, from 0 to 5 min. Next, gradient elution was applied from 5 to 30 min, gradually changing from 85% A and 15% B to 0% A and 100% B. This was followed by isocratic conditions from 30 to 35 min, maintaining 0% A and 100% B. The injection volume was 10 μL, and the flow rate was 1 mL/min [22]. By matching the retention durations of the detected peaks with the reference compounds, phenolic compounds were identified. The corresponding concentrations were derived by creating standard curves for each compound, using several concentrations of equivalent standards.

### 2.6. Animals

A total of 28 healthy adult male SD rats (*Rattus norvegicus*) (6–7 weeks old, 180 ± 5 g) were acquired from the Animal House of Beirut Arab University, Beirut, Lebanon. The sample size was selected based on the number of animals required to achieve a good statistical power while maintaining ethical consideration. Animals were housed in well-ventilated conditions (humidity 60–70% and temperature 23 ± 2 °C) with a photoperiod of 12 h light/12 h dark with water and food ad libitum. Humane handling was given to every laboratory animal, in compliance with the recommendations of the National Institutes of Health (NIH). All animals were monitored adequately during the whole study to ensure that the animals do not experience any pain or distress that exceeds the level specified by the Research Ethics Committee “Institutional Review Board” (IRB) of Beirut Arab University, Beirut, Lebanon (with approval IRB protocol number: 2023-A-0050-S-P-0513).

### 2.7. Experimental Design and Sample Collection

Animals were distributed randomly into 7 groups (*n* = 4 per group) and treated daily by oral gavage for 60 days. The duration of spermatogenesis in rats lasts between 51.6 and 56 days [23]. Group 1 (control): Ctrl, treated with normal saline. Group 2: LS, treated with 100 mg/kg *Lepidium sativum* extract. Group 3: OM, treated with 100 mg/kg *Origanum majorana* extract. Group 4: FH, treated with 100 mg/kg *Ferula hermonis* extract. Group 5: ES, treated with 100 mg/kg *Eruca sativa* extract. Group 6: MIX, treated with a mixture of the above-mentioned plants. Group 7 (positive control): VitE, treated with 100 mg/kg of vitamin E. Animal treatment, sample collection, and data analysis were performed in a blinded manner to avoid bias, where investigators were unaware of group assignments during these stages. Blinding was only removed after all data had been gathered and examined to ensure that the results were unaffected by prior knowledge of the groups. The choice of doses for the experimental aqueous herbal extracts was established based on previous experiments [19,24,25,26,27]. Besides, neither the exposure doses selected for the present work caused pain or any signs of mortality to the animals, nor were further adverse effects detected among the experimental groups; therefore, no animals, experimental units, or data points were excluded from the experiment or data analysis. Throughout the experiment, body weight (BW) was measured every other day, and the rats were monitored daily for water and food intake.

After 60 days of treatment, rats were sacrificed, and blood specimens were collected by heart puncture. Testes were removed by open castration, and their weights were determined. Caudal epididymal semen samples were also collected. The gonadosomatic index (GSI) was calculated by dividing the BW by the testes weight and multiplying by 100. The left testes were preserved in Bouin’s fixative for histological examination, while the right testes were snap frozen, stored at −80 °C, and then homogenized, and protein concentration were quantified using the Bradford method [28]. Testes homogenates were used for further biochemical and molecular analysis. The primary outcome measure was spermiogram (sperm count, motility, and vitality). Secondary outcomes included sex hormones (testosterone and LH), lipid peroxidation (MDA) and antioxidant markers (SOD and GSH), histomorphometric measurements of testicular seminiferous tubules, and molecular markers related to key signaling pathways involved in cellular growth, survival, and stress response (AKT, mTOR, PTEN, and vimentin).

### 2.8. Sperm Analysis

Following the sacrifice, the right epididymis was removed. Sperm count was determined using a hemocytometer based on the technique described by Prasad et al. [29]. Additionally, the sperm motility and vitality were assessed using a light microscope according to the protocols established by the World Health Organization [30] and Didion et al. [31], respectively.

### 2.9. Serum Hormone Analysis

Serum luteinizing hormone (LH) and testosterone levels were measured by Elecsys assay kits (Roche, Munich, Germany) using an electrochemiluminescence immunoassay analyzer (Cobas e411, Roche, Germany).

### 2.10. Testes Lipid Peroxidation and Antioxidant Markers

Using right teste homogenates, superoxide dismutase (SOD) was assessed based on the procedure detailed by Giannopolitis and Ries [32], reduced glutathione (GSH) using the procedure proposed by Moron et al. [33], and malondialdehyde (MDA), a marker of lipid peroxidation, following the protocol described by Heath and Packer [34].

### 2.11. Histological Examination and Morphometric Evaluation

For histological examinations, segments of each animal’s left testis were fixed in formalin fixative, dehydrated in increasing concentrations of ethanol solutions, cleared with xylene, embedded in paraffin, and sectioned at 5 μm using a rotary microtome (Leica RM2235, Leica Microsystems, Deerfield, IL, USA). The slices were hematoxylin and eosin (H&E) stained and assessed using a Leica DM500 with ICC50 W CAM (Leica Microsystems, Deerfield, IL, USA). Image J version 1.50i was employed to morphometrically evaluate the diameter and epithelial height of 20 arbitrarily selected STs. Sertoli cells, Leydig cells, and spermatogonia were enumerated in 20 STs and intertubular spaces from 10 distinct fields, each containing 100 STs, that were designated haphazardly [35,36]. The evaluation of testicular biopsy score was performed in 10 tubules that were arbitrarily selected, as defined by the procedure described by Johnsen [37]. The study of the morphometric parameters was achieved in two separate sections for each animal, and the average per animal was then calculated.

### 2.12. Western Blotting Assay

Proteins with equivalent volumes were separated on a 10% SDS-PAGE gel and then electroblotted onto a polyvinylidene difluoride (PVDF) membrane. Blocking of the membranes was done using 5% nonfat milk after a rinse in 1× TRIS buffered saline (TBS). After an overnight incubation with primary antibodies against the target proteins (AKT, mTOR, PTEN, and vimentin), the membranes were rinsed and incubated with a horseradish peroxidase (HRP)-conjugated secondary antibody. The membranes were washed, and an enhanced chemiluminescence (ECL) substrate was used for evaluation. All immunoblots were normalized to glyceraldehyde-3-phosphate dehydrogenase (GAPDH), where fold changes in protein expression were determined and the band intensities of the blot were quantified by Image J Software (version 1.54g, Wayne Rasband and Contributors National Institutes of Health, Kensington, MD, USA).

### 2.13. Statistical Analysis

Using SPSS version 24 and one-way analysis of variance (ANOVA), differences between groups were statistically assessed. F values with *p* < 0.05 were considered significant with a 95% confidence interval. The data are reported as mean ± standard error of the mean (SEM).

## 3. Results

### 3.1. Total Phenolic Content of the Plant Extracts

**Figure 1:**
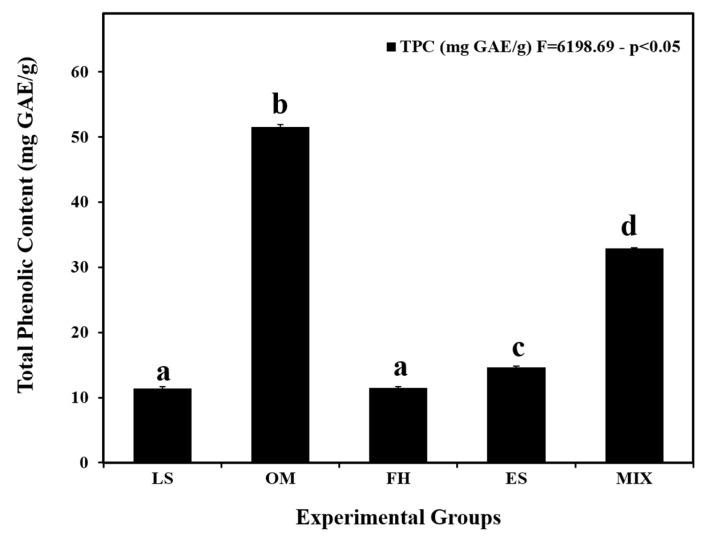
Total phenolic content of the plant extracts. Data are presented as means ± SEM. Error bars indicate standard error of the mean (SEM). Means with different letters are significantly different (*p* < 0.05). LS, *Lepidium sativum* aqueous extract; OM, *Origanum majorana* aqueous extract; FH, *Ferula hermonis* aqueous extract; ES, *Eruca sativa* aqueous extract; MIX, plant mix. Superscript letters (a, b, c, and d) indicate significance within groups. The values marked with the same superscript letter are similar (*p* > 0.05), whereas those marked with different ones are significantly different (*p* < 0.05).

The plant extracts’ TPC was determined (Figure 1). The total phenolic content of OM was the highest, ~51.56 ± 0.32 mg GAE/g, and significantly different than the other plant extracts (*p* < 0.05). The second highest TPC was measured in MIX, 32.94 ± 0.06 mg GAE/g, followed by ES, 14.65 ± 0.16 mg GAE/g, with significance between the two extracts (*p* < 0.05). The extracts LS and FH had the lowest total phenolic content, 11.42 ± 0.25 and 11.47 ± 0.22 mg GAE/g, respectively, without significant difference between the two extracts (*p* > 0.05).

### 3.2. Antioxidant Activity of the Plant Extracts

**Figure 2:**
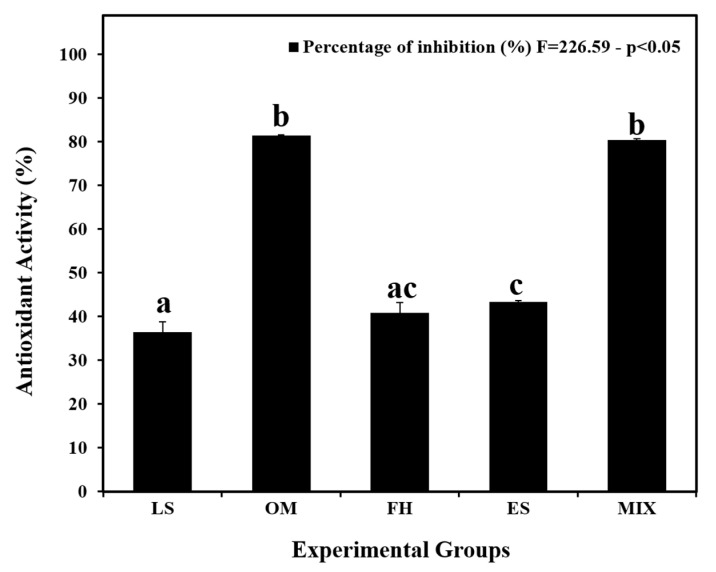
Antioxidant activity of the plant extracts. Data are presented as means ± SEM. Error bars indicate standard error of the mean (SEM). Means with different letters are significantly different (*p* < 0.05). LS, *Lepidium sativum* aqueous extract; OM, *Origanum majorana* aqueous extract; FH, *Ferula hermonis* aqueous extract; ES, *Eruca sativa* aqueous extract; MIX, plant mix. Superscript letters (a, b, and c) indicate significance within groups. The values marked with the same superscript letter are similar (*p* > 0.05), whereas those marked with different ones are significantly different (*p* < 0.05).

The plant extracts’ antioxidant activity was studied using the DPPH test to estimate their free-radical scavenging aptness (Figure 2). The OM and MIX extracts displayed the highest antioxidant activities, reaching 81.47 ± 0.15% and 80.39 ± 0.30%, respectively, with an insignificant difference between them (*p* > 0.05). The ES and FH extracts followed, exhibiting antioxidant activities of 43.33 ± 0.30% and 40.81 ± 2.37%, respectively, with an insignificant difference between them (*p* > 0.05). The LS extract exhibited the lowest antioxidant activity, measuring 36.44 ± 2.29%, with no significant difference compared to FH (*p* > 0.05).

### 3.3. Identification and Quantification of Phenolic Compounds by HPLC

**Figure 3:**
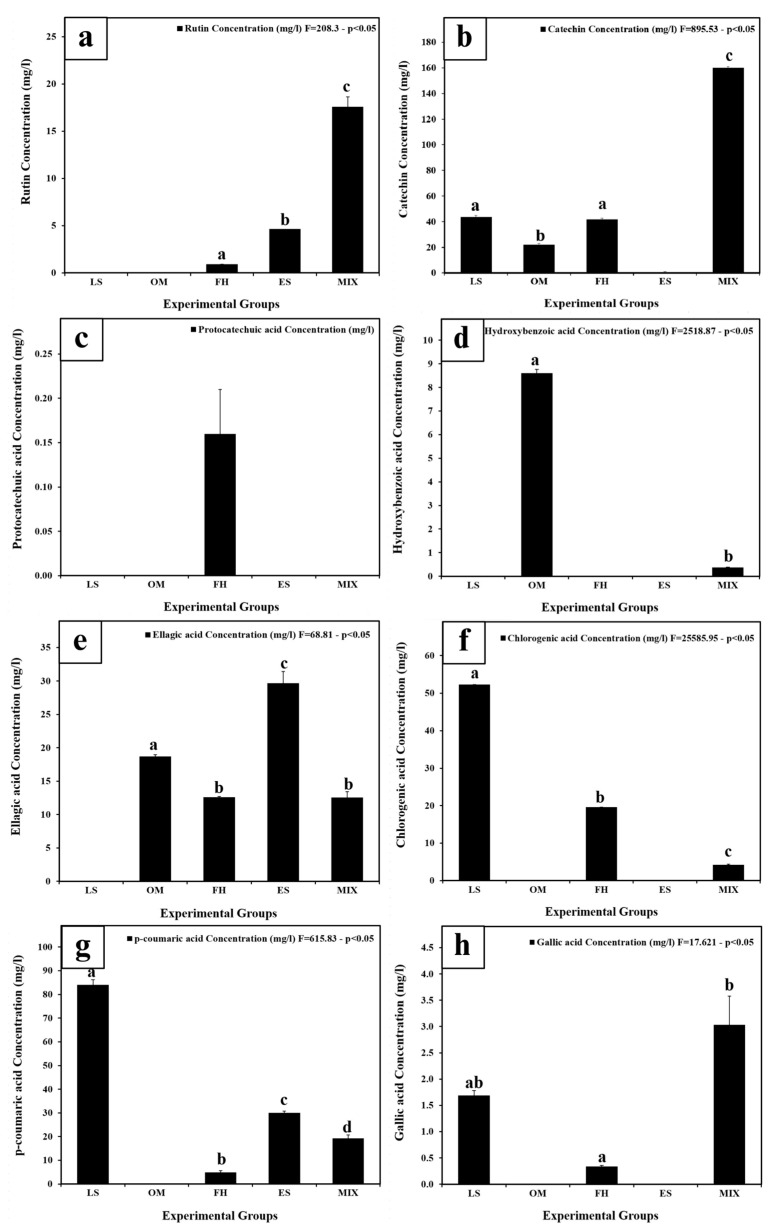
Identification and quantification of the main polyphenols of plant extracts. (**a**) Rutin Concentration (mg/l). (**b**) Catechin Concentration (mg/l). (**c**) Protocatechuic acid Concentration (mg/l). (**d**) Hydroxybenzoic acid Concentration (mg/l). (**e**) Ellagic acid Concertation (mg/l). (**f**) Chlorogenic acid Concentration (mg/l). (**g**) p-coumaric acid Concentration (mg/l). (**h**) Gallic acid Concentration (mg/l). Data are presented as means ± SEM. Means with different letters are significantly different (*p* < 0.05). LS, *Lepidium sativum* aqueous extract; OM, *Origanum majorana* aqueous extract; FH, *Ferula hermonis* aqueous extract; ES, *Eruca sativa* aqueous extract; MIX, plant mix. Superscript letters (a, b, c, and d) indicate significance within groups. The values marked with the same superscript letter are similar (*p* > 0.05), whereas those marked with different ones are significantly different (*p* < 0.05).

Phenolic compounds found in various plant extracts were identified and evaluated using HPLC (Supplementary Material, Figures S1 and S2). Eight distinct types of polyphenols, namely ellagic acid, rutin, catechin, protocatechuic acid, hydroxybenzoic acid, chlorogenic acid, *p*-Coumaric acid, and gallic acid, were recognized and measured as illustrated in Figure 3a–h. Rutin was the most abundant in the MIX (17.58 ± 1.04 mg/L), followed by significantly lower amounts in ES (4.63 ± 0.00 mg/L) and FH (0.91 ± 0.01 mg/L) (*p* < 0.05) (Figure 3a). Catechin was detected at the highest concentration of all phenolic compounds in the MIX (160.11 ± 2.61 mg/L), while in the other extracts, the load of catechin was significantly lower (*p* < 0.05). It decreased as follows: followed by LS 43.64 ± 2.47 mg/L, FH 41.70 ± 0.00 mg/L, and OM 22.04 ± 2.17 mg/L (Figure 3b). FH was found to be the only plant extract that contained protocatechuic acid, with a concentration of 0.16 ± 0.05 mg/L. (Figure 3c). Hydroxybenzoic acid was found in OM at 8.61 ± 0.16 mg/l, with a significantly lower amount in MIX (0.37 ± 0.01 mg/L) (Figure 3d). Ellagic acid was most abundant in ES with a concentration of 29.69 ±1.72 mg/L. OM had the next highest concentration at 18.69 ± 0.27 mg/L, and there was a significant difference between the two (*p* < 0.05) as shown in Figure 3e. FH and MIX had similar concentrations of ellagic acid, with 12.59 ± 0.11 mg/L and 12.51 ± 0.85 mg/L, respectively; however, an insignificant difference was detected between the two (*p* > 0.05). The concentration of chlorogenic acid was found to be highest in LS at 52.36 ± 0.00 mg/L, followed by FH at 19.64 ± 0.00 mg/L and MIX at 4.18 ± 0.26 mg/L, with significant differences observed between them (*p* < 0.05) as shown in Figure 3f. *p*-Coumaric acid was most abundant in LS 84.06 ± 2.12 mg/L, followed by ES 30.06 ± 0.63 mg/L, MIX 19.22 ± 1.49 mg/L, and FH 4.91 ± 0.77 mg/L (Figure 3g), with significant differences between the extracts (*p* < 0.05). The highest concentration of gallic acid was found in MIX, with a value of 3.03 ± 0.55 mg/L, followed by LS, with a value of 1.69 ± 0.09 mg/L. Notably, an insignificant difference was observed between MIX and LS (*p* > 0.05). FH had the lowest concentration of gallic acid, with a value of 0.34 ± 0.02 mg/L, and an insignificant difference was observed between FH and LS (*p* > 0.05).

### 3.4. Total Body Weight, Testes Weight, and Gonadosomatic Index

During the experiment phase, the body weights of the control and experimental groups did not differ significantly (*p* > 0.05) (Table 1). However, the extracts of OM, ES, and VitE significantly increased testicular weight compared to rats in the control group and the other experimental groups. However, in the OM group, the gonadosomatic index was revealed to be significantly higher than in the control group.

### 3.5. Serum Hormonal Profile

**Figure 4:**
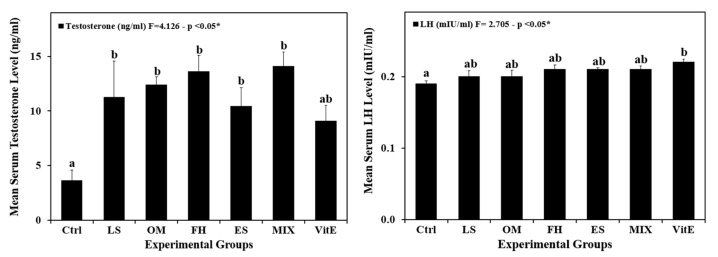
Changes in serum testosterone (ng/mL) and luteinizing hormone (LH) (mIU/mL) levels among the experimental groups. Data are presented as mean ± SEM (n = 4/group). Error bars indicate standard error of the mean (SEM). Different letters indicate significant differences between groups (*p* < 0.05, one-way ANOVA followed by Tukey’s post hoc test). Ctrl, normal control; LS, *Lepidium sativum* aqueous extract-treated; OM, *Origanum majorana* aqueous extract-treated; FH, *Ferula hermonis* aqueous extract-treated; ES, *Eruca sativa* aqueous extract-treated; MIX, plant mix-treated; VitE, vitamin E-treated. Superscript letters (a and b) indicate significance within groups. The values marked with the same superscript letter are similar (*p* > 0.05), whereas those marked with different ones are significantly different (*p* < 0.05). * Indicates significance at *p* < 0.05.

Figure 4 reveals a highly significant increase (*p* < 0.001) in serum testosterone levels in the LS, OM, FH, ES, and MIX groups after 60 days of treatment with plant extracts compared with the control. On the other hand, a lack of significant differences (*p* > 0.05) in serum LH concentration was detected between the control and plant-treated groups. There was only a significant difference between the VitE and the control groups.

### 3.6. Sperm Parameters Analysis

Epididymal sperm concentration demonstrated a significant increase (*p* < 0.001) in rats subjected to OM, FH, and MIX compared to the control. No further significant alterations were observed in sperm concentration in other experimental groups, nor in sperm motility and vitality of all groups as shown in Table 2. Similarly, the mean Johnsen’s score in all groups did not show any significant differences from the control group (*p* > 0.05).

### 3.7. Changes in Testes Oxidative Stress Markers

As shown in Table 3, testicular GSH levels in all groups were significantly higher than those in the control, with the most significant enhancement (*p* < 0.001) in the groups treated with OM, ES, and MIX. Compared with control, insignificant differences were reported in the levels of SOD and MDA in the testes of the experimental groups.

### 3.8. Histology and Morphometric Measurements

**Figure 5:**
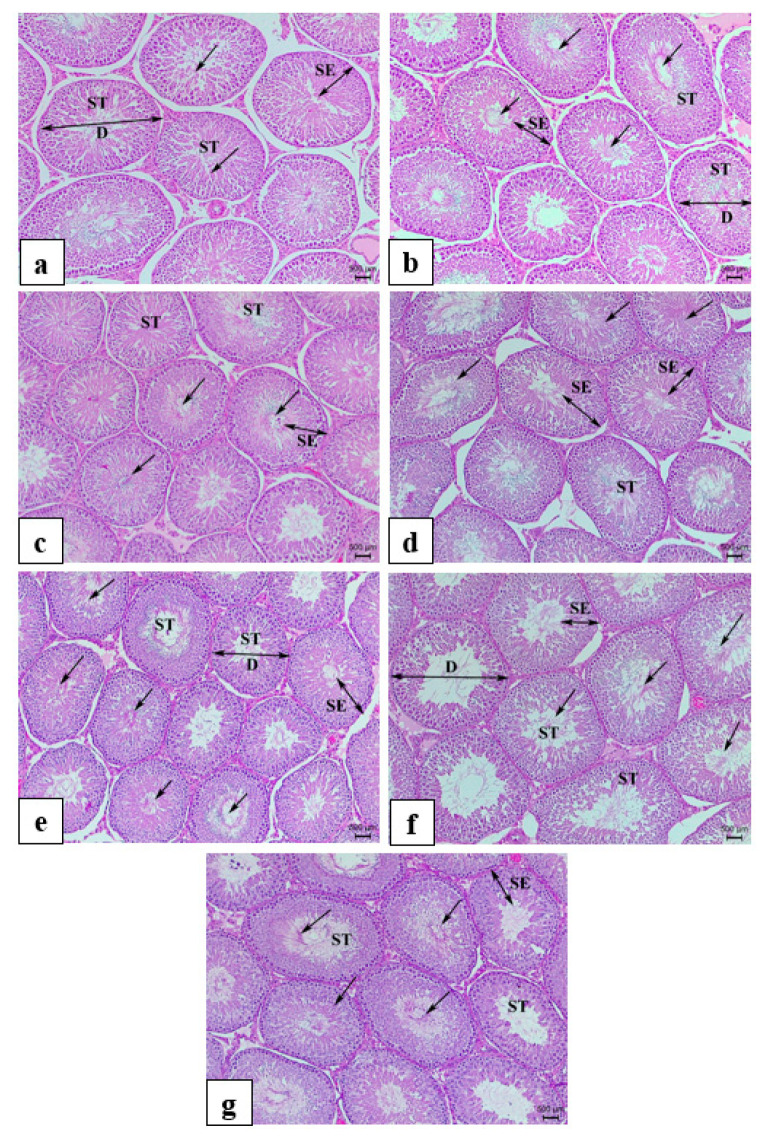
Light micrograph showing representative H&E-stained seminiferous tubules of testicular tissue of (**a**) Ctrl, normal control; (**b**) LS, *Lepidium sativum* aqueous extract-treated; (**c**) OM, *Origanum majorana* aqueous extract-treated; (**d**) FH, *Ferula hermonis* aqueous extract-treated; (**e**) ES, *Eruca sativa* aqueous extract-treated; (**f**) MIX, plant mix-treated; (**g**) positive control, VitE, vitamin E-treated. (Magnification: ×100; scale bar: 500 μm). Arrow: degree of spermatogenesis and sperm cells in the lumen ST: seminiferous tubules; small double-sided arrow: thickness of SE. SE: seminiferous epithelium height; big double-sided arrow: full diameter D of ST, Lyd: Leydig cells; spg: spermatogonia cells; ser: Sertoli cells.

**Figure 6:**
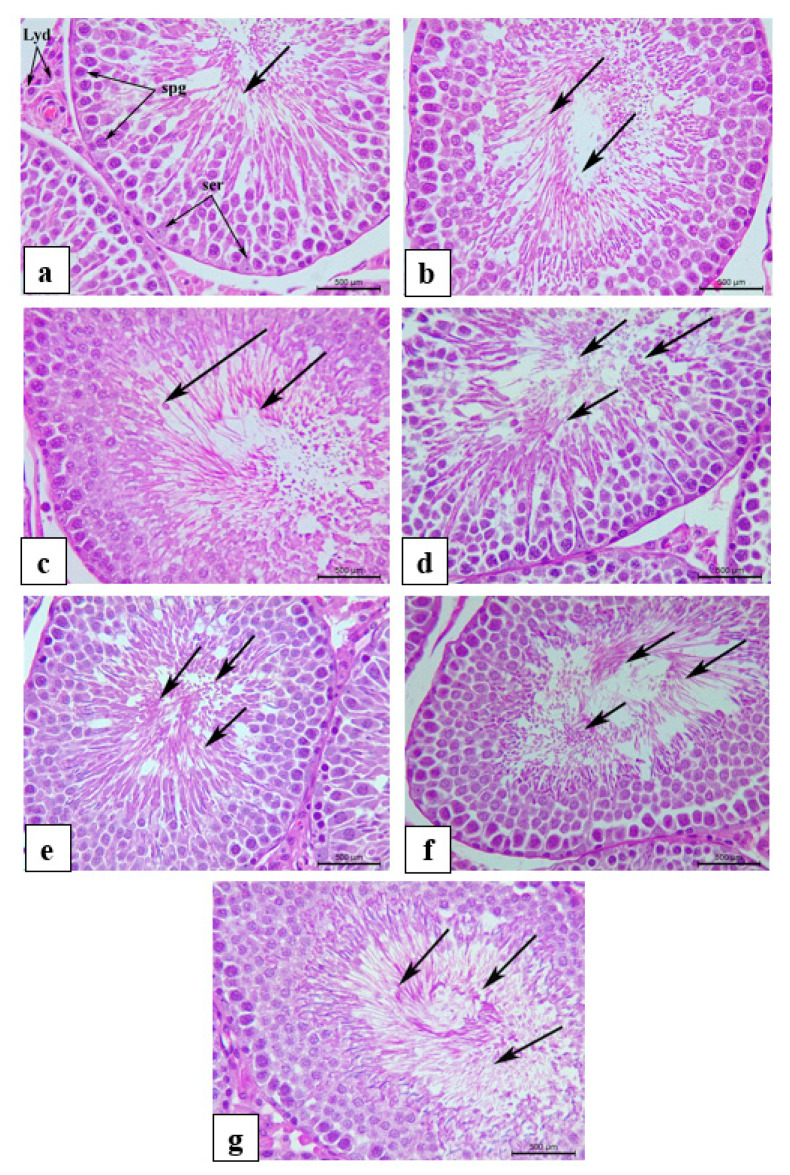
Light micrograph showing representative H&E-stained seminiferous tubules of testicular tissue of (**a**) Ctrl, normal control; (**b**) LS, *Lepidium sativum* aqueous extract-treated; (**c**) OM, *Origanum majorana* aqueous extract-treated; (**d**) FH, *Ferula hermonis* aqueous extract-treated; (**e**) ES, *Eruca sativa* aqueous extract-treated; (**f**) MIX, plant mix-treated; (**g**) positive control, VitE, vitamin E-treated. (Magnification: × 400; scale bar: 500 μm). Arrow: complete spermatogenesis and sperm cells in the lumen; Lyd: Leydig cells; spg: spermatogonia cells; ser: Sertoli cells.

**Figure 7:**
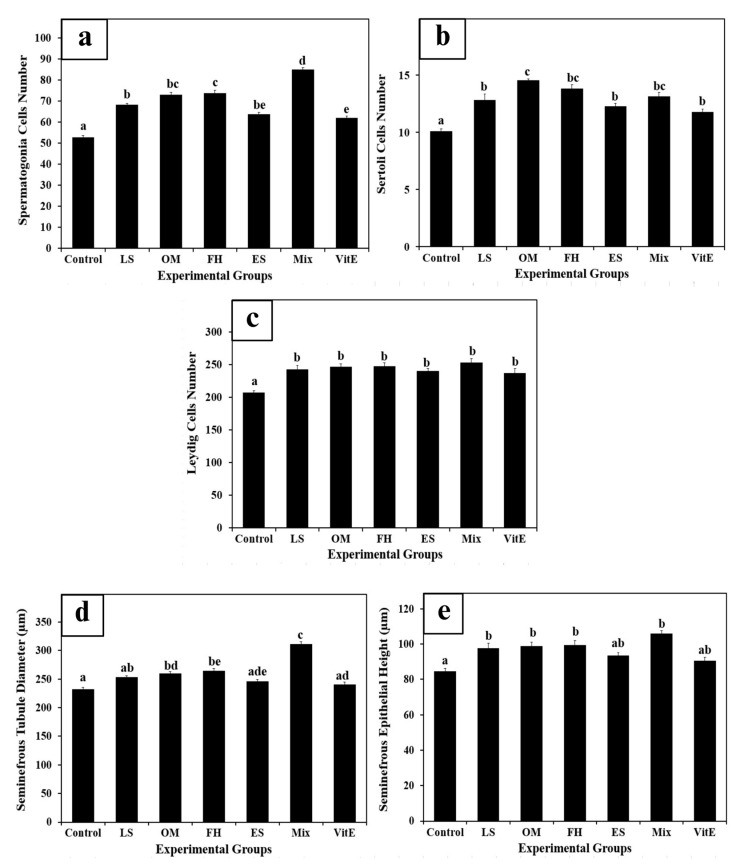
Morphometric measurements of means of (**a**) spermatogonia cell count; (**b**) Sertoli cell count; (**c**) Leydig cell count; (**d**) seminiferous tubules’ diameter; and (**e**) seminiferous epithelial thickness. Data are presented as means ± SEM (n = 4/group). Means with different letters are significantly different. Values with *p* < 0.05 are significant, n = 4/group. Ctrl, normal control; LS, *Lepidium sativum* aqueous extract-treated; OM, *Origanum majorana* aqueous extract-treated; FH, Ferula hermonis aqueous extract-treated; ES, *Eruca sativa* aqueous extract-treated; MIX, plant mix-treated; VitE, vitamin E-treated. Superscript letters (a, b, c, d, and e) indicate significance within groups. The values marked with the same superscript letter are similar (*p* > 0.05), whereas those marked with different ones are significantly different (*p* < 0.05).

Histological observation did not reveal any pathological alterations in the testis light micrographs of H&E sections among the control and experimental groups. Moreover, normal seminiferous tubular architecture was manifested with no morphological changes in the structure and arrangement of the seminiferous tubular epithelial cells and interstitial tissue in the experimental groups compared to those in the Ctrl and VitE groups (Figure 5 and Figure 6). Fertility has been linked to sperm production. Here, in addition to the significant improvement in testosterone hormonal levels, the generation of sperm and other aspects of testicular quality were the main concerns. Therefore, the quality of the testes was determined by counting the number of spermatogonia, Leydig, and Sertoli cells and measuring the thickness of the germinal epithelium as well as the diameter of the seminiferous tubules. Quantitative data obtained from histological morphometric measurements (Figure 7a–e) exhibited a significant orderly increase in the count of spermatogonia and Leydig cells, as well as seminiferous tubular diameter and germinal epithelial thickness in all the experimental groups, with the highest values observed in MIX, followed by FH, OM, LS, and ES, as well as positive control VitE, when compared to Ctrl. Furthermore, the data showed a significant incremental change in the count of spermatogonia cells and Leydig cells, as well as the diameter and the epithelial height in seminiferous tubules of MIX, respectively. Although an incremental change in the diameter and epithelial height thickness of seminiferous tubules was observed in the positive control VitE, it did not significantly differ from that of Ctrl. Figure 7b indicates that the mean Sertoli cell counts of all experimental and positive control groups were significantly different from those of the control group, where FH, OM, and MIX attained the highest significant values compared to the other experimental groups.

### 3.9. Western Blot Analysis

**Figure 8:**
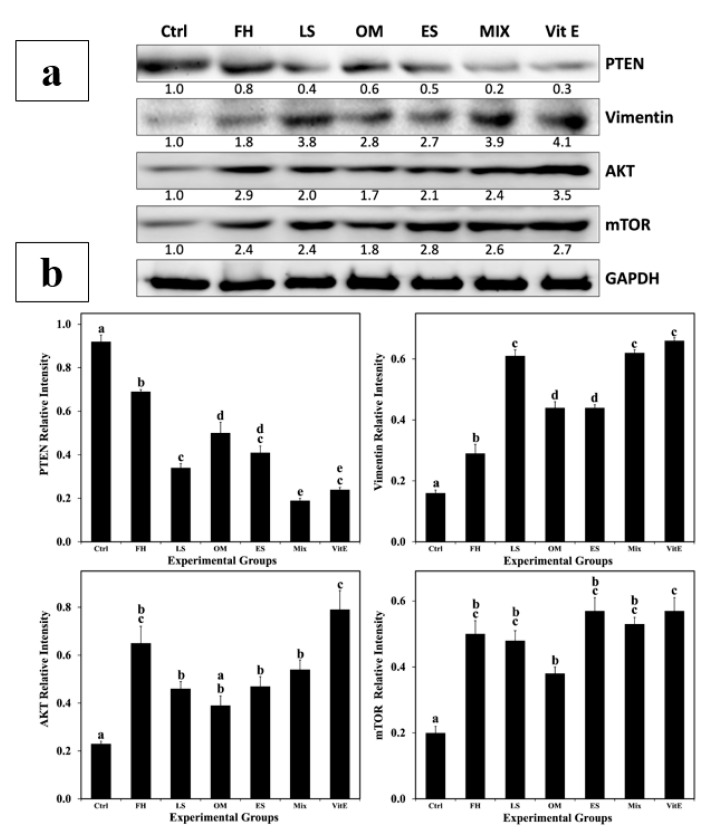
Expressions of PTEN, vimentin, AKT, and mTOR proteins in male rats treated with folk herbs or vitamin E. Animals are either treated with the aqueous extract of *Ferula hermonis* (FH), *Lepidium sativum* (LS), *Origanum majorana* (OM), *Eruca sativa* (ES), mix of plant extracts (MIX), or vitamin E (VitE). (**a**) Western immunoblots showing protein expression. Band intensities were normalized to their respective GAPDH bands. Fold changes in protein expressions compared with the untreated control samples (Ctrl) are represented below each protein band. (**b**) Relative protein band intensities. Superscript letters (a, b, c, d, and e) indicate significance within groups. The values marked with the same superscript letter are similar (*p* > 0.05), whereas those marked with different ones are significantly different (*p* < 0.001).

The signaling mechanisms involved in spermatogonia stem cell proliferation and self-renewal were analyzed (Figure 8). According to Western blot analysis, the expression levels of vimentin were elevated in the testes of treated rats compared to controls, especially in the MIX- and VitE-treated animals, where the intensity analysis of antibody staining of the proteins bound to the membrane increased by 3.9 and 4.1-fold, respectively. The expression levels of AKT and mTOR were also, hand in hand, upregulated in the testes of experimental animals, with the highest fold change noted for AKT being in the FH and VitE groups (2.9 and 3.5) and for mTOR in the ES and VitE groups (3.1), indicating that the AKT/mTOR pathway was activated. These upregulations were accompanied by a downregulation of PTEN expression in the testes of treated animals, with the lowest fold change recorded in the MIX group (0.2).

## 4. Discussion

Even with recent medical progress in male impotence treatment, some men favor botanical products as a substitute, given that many herbs have the ability to improve sexual potential and bypass the adverse effects of the compounds used to increase sperm count or to enhance libido [15]. Previous studies have identified the positive effect of four regional plants, *Lepidium sativum*, *Origanum majorana*, *Ferula hermonis*, and *Eruca sativa*, on improving male fertility in several animal species. Saleh et al. [38] demonstrated that providing OM supplements to Bovans brown laying hens improved their ovarian follicular development, antioxidant activity, hormonal state, steroidogenesis, and productive performance. Another study on LS extract demonstrated that lower doses (62.5–250 µg/mL) improved cell viability, membrane integrity, and antioxidant activity in TM3 Leydig cells in mice [39]. In addition, the effectiveness of FH seed oil in treating erectile dysfunction was demonstrated by Ayuob et al. [40]. The reproductive effect of ES was also investigated by Hussein F. Zeina [41] on male albino mice, where the results showed improvements in sperm activity and testosterone level, with decreased sperm mortality and abnormalities. Hence, this study aims to evaluate the effects of these selected regional plants on male SD rat fertility parameters, focusing mainly on spermatogenesis.

Bearing in mind the importance of polyphenols in defending cellular components against oxidative damage ensuing from the overproduction of free radicals, there is an eminent interest in exploring the difference in the TPC among plant extracts. As indicated by the findings of Roby et al., the OM leaves were exposed to possess the utmost extractable phenolic compounds (5.20 mg GAE/g), which aligns with our findings showing that OM had the highest TPC [42]. The aerial parts of OM were evaluated for TPC by Baatour et al., who reported a value of 8.86 mg GAE/g, which is lower than the TPC for OM observed in our results [43]. A study on ES found its seeds to be rich in phenolics, with a concentration of 27.1 ± 0.2 mg GAE/g, and reported that the leaves and flowers contained 23.07 ± 0.11 and 19.9 ± 0.3 mg GAE/g, respectively—both higher than the values obtained in our study [44]. In an investigation led by Kasabe et al. [45], the methanolic extract of LS seeds had a TPC of 21 mg GAE/g, also higher than our study’s TPC for LS. Additionally, Abdel-Kader et al. [46] stated a total phenolic content of 73.6 mg/g in the FH root extract, which exceeded the TPC of FH in our study. The level of phenolic content in plants may diverge prominently depending on the plant species and its numerous parts, as they might enclose fluctuating quantities of bioactive compounds. On top of this, variations in the level of bioactive components in natural elements may occasionally be associated uniquely with agro-climatic circumstances [47]. Moreover, disparities in TPC amounts can be linked to variations in the extraction protocol and the solvent used.

The DPPH test was employed to evaluate the plant extracts’ capacity to scavenge free radicals and demonstrate antioxidant activity. The attained results are in accordance with the study led by Dhull et al. [47], precisely with the findings obtained via the application of the DPPH colorimetric evaluation. The outcomes specified that extracts with prominent phenolic content (OM and MIX) displayed greater antioxidant potential and better suppression of free radicals.

The HPLC analysis effectively identified and quantified polyphenols in plant extracts, revealing significant compounds with known health benefits, such as antioxidative, antihypertensive, immunomodulatory, anticancer, and antimicrobial effects [48]. In our study, LS was found to contain catechin [49], chlorogenic acid [50], *p*-coumaric acid [51], and gallic acid [52], consistent with other research findings. Similarly, the OM extract was found to contain catechin, ellagic acid, and hydroxybenzoic acid, aligning with previous studies, except for ellagic acid [43,53]. For FH, the presence of ellagic acid, rutin, catechin, protocatechuic acid, chlorogenic acid, *p*-coumaric acid, and gallic acid was observed, in agreement with a previous study that identified rutin, catechin, and protocatechuic acid [54]. In the case of ES, rutin, ellagic acid, and *p*-coumaric acid were detected, consistent with prior studies, though ellagic acid had not been previously reported [24,55]. Variations in compound identification may be attributed to differences in the plant extract origins, solvents, and extraction methods used.

Rutin, highest in MIX, supports male infertility treatment due to its anti-apoptotic, antioxidant, and anti-inflammatory effects, protecting the male reproductive system from inflammation, oxidative stress, and apoptosis [56]. Catechin, also highest in the MIX, may be elevated due to a synergistic effect among the extract components [57,58]. As detailed by Rai et al. [59], catechin may enhance male sexual function by improving intromission and mounting, reducing latencies, and supporting reproductive health. Hydroxybenzoic acid, known for its antioxidant properties, was found in higher concentrations in the OM extract than in the MIX. Its antioxidant activity is linked to its ability to chelate reactive oxygen species (ROS), preventing lipid peroxidation and inhibiting pro-oxidant enzymes [60]. Ellagic acid, a polyphenol known for its cancer-fighting properties [61], was found in the highest concentration in the ES extract. It plays a vital role in male reproductive health [62] by protecting sperm DNA from oxidative stress and regulating male infertility through its antioxidant, anti-inflammatory, and anti-genotoxic properties [63]. Chlorogenic acid, highest in the LS extract, exhibits antioxidant properties by inhibiting oxidative damage [64]. Chlorogenic acid also improves male reproductive health by protecting against reproductive toxicity, testicular inflammation, oxidative stress, and apoptosis caused by various factors [65,66]. *p*-Coumaric acid, which exhibits a range of pharmacological effects [67], was found in the highest amounts in LS extract. Its antioxidant properties help protect the male reproductive system by reducing oxidative stress and inflammation [68]. Lastly, the MIX had the highest concentration of GA, which is known for its antioxidant, anti-inflammatory, and protective effects on the male reproductive system, protecting it from reproductive toxicity caused by toxins [69,70]. The study results indicate that the diverse phenolic compounds in the extracts enhance antioxidant activity and support the male reproductive system.

During the present investigation, treatment with different plant extracts did not affect total body weight. However, plant administration increased testes weight and gonadosomatic index, especially in the OM group. These findings align with previous research reported by El-Wakf et al. [13], where OM increased the testes weight. The structural and physiological functions of the epididymis, testes, and other reproductive organs depend on testosterone and other androgens [71]. In this study, the significant increase in testosterone levels in all experimental groups could be responsible for the enhancement of the testes’ somatic and germinal cells, including their quantity and role, which would, in turn, increase the testes weight and ratio [72]. Plant extracts also significantly increased epididymal sperm concentrations in all groups. This observation aligns with the hypothesis that elevated blood testosterone levels are correlated with increased sperm production [73]. The results of the present study are in accordance with those described by Razaq et al. [12], where it was demonstrated that LS was able to enhance testosterone levels in mice injected with aqueous extract. LH plays a pivotal role in stimulating Leydig cells to produce testosterone; however, in the present study, the increase in LH levels in rats administered plant extracts was not found to be significant [5].

In the experimental groups, seminiferous tubular epithelial cells and interstitial tissues maintained their normal morphology and displayed a typical seminiferous tubular architecture. Additionally, in the present investigation, plant extracts significantly increased spermatogonia, Sertoli, and Leydig cell counts, seminiferous tubule diameter, and seminiferous epithelial height in all treatment groups. In fact, spermatozoa concentration in the ejaculate as well as reproductive effectiveness can be directly correlated with the quantitative histology of the testicles, Leydig and Sertoli cell effectiveness, and general morphometry of the seminiferous tubule [74]. Furthermore, the increased ability of Leydig cells to produce testosterone, particularly in the metabolic pathway after cholesterol, may be accountable for the observed increase in testosterone levels [75]. Furthermore, according to Olawuyi et al. [76], the ST epithelium can positively affect the rate of sperm production. It has also been reported that when the spermatogenesis rate increases, the thickness and diameter of STs also increase [74]. These findings are in accordance with previous results, where *Punica granatum* L. extract increased both ST diameter and epithelial thickness in albino Wistar rats exposed to heat [77].

Redox imbalance or overproduction of reactive oxygen species (ROS) plays a role in disease pathophysiology and reproductive damage [78]. Free radical overproduction or a deficiency in antioxidants such as SOD and GSH leads to the loss of cellular redox equilibrium, which elevates MDA levels that, in turn, react with molecules to cause oxidative stress [79]. Precisely, testes that are rich in polyunsaturated fatty acids are more prone to oxidative stress and lipid peroxidation [80]. Studies have demonstrated that medicinal herbs possess strong antioxidant effects [81]. The non-enzymatic antioxidant defense mechanism of an organism relies mainly on GSH [82]. Because of its role in cell metabolism and reducing abilities, GSH plays a significant part in sustaining the integrity of cells and is a direct scavenger of free radicals [83]. It was also established that GSH possesses the ability to improve a broad spectrum of fertility parameters, including sperm motility and seminiferous tubule morphology [84]. The increase in testes GSH, detected during this study, particularly in the OM, ES, and MIX groups, can be explicated by the existence of natural antioxidants in plants that can stimulate antioxidants or scavenge free radicals [85]. Likewise, El-Wakf et al. [14] reported that GSH levels increased in rats’ testes after the administration of OM oil. Regarding the enzymatic antioxidant SOD, El-Wakf et al. [14] exposed that OM improved its levels in the testes, but the increase documented in the present experimental groups was not statistically significant. In opposition, β-carotene has been stated to alleviate testes oxidative stress by limiting lipid peroxidation and returning SOD and GSH to normal physiological levels [86]. The plant extracts, in the current investigation, caused no discrepancies in testicular MDA levels, suggesting that they did not engender lipid peroxidation. These outcomes are aligned with Grami et al. [87], who reported that ES did not modify MDA levels in the testes.

The PI3K/AKT/mTOR signaling pathway is recognized to regulate the cell cycle, protein synthesis, and metabolism of cell energy [88]. PI3Ks represent an important family of lipid kinases [89]. Serine-threonine kinase AKT is a vital PI3K downstream protein. It can be identified in Sertoli and spermatogenic cells. It is also the upstream regulator in charge of sperm cell homeostasis, which can uphold cell balance and proliferation while unveiling anti-apoptotic action [7]. The signal integrator of great importance in the PI3K/AKT/mTOR signaling network, mTOR, regulates metabolism, cell growth, and proliferation [90]. Controlling Sertoli cell metabolic activities and redox balance, as well as SSC development and preservation, is importantly under the guidance of mTOR [6]. This was in accordance with the results of the present study, in which AKT and mTOR levels were clearly enhanced in all experimental groups. Similarly, seed extracts of *Plantago asiatica* L. were found to lessen the damage to the reproductive system of male rats produced by nonylphenol by moderating the PI3K/AKT/mTOR pathway [91]. Overexpression of PTEN can prevent AKT activation; thus, PTEN negatively controls the PI3K signaling pathway [7]. PTEN was downregulated in all groups treated with the experimental plants under examination. These findings are in agreement with previous studies, where ferulic acid, found in herbs such as female ginseng, was revealed to lessen testicular oxidative stress by inhibiting PTEN [92].

Former research has exposed that Sertoli cell vimentin filaments, an indispensable component of the cytoskeleton, are of foremost importance for sustaining spermatogenesis [93]. Impairment of the testes, vacuolation, and detachment of spermatogenic cells may arise from Sertoli cell vimentin filaments disintegrating away from the cell membrane. Because of the lack of Sertoli cell sustenance, these sloughing cells may undergo apoptosis [94]. The deductions of this study show a vigorous correspondence between increased vimentin expression and upsurges in Sertoli cell number and epithelial thickness in testicular seminiferous tubules, particularly in the MIX group. The notable increase in the Sertoli cell calculation suggests that amplified vimentin expression may possibly enhance the development of the aforementioned cells. These outcomes are consistent with those of a previous investigation that connected vimentin with cellular multiplication and differentiation [95]. Furthermore, the results also line up with those published by Yang et al. [8], where an intensification in vimentin expression levels and spermatogenesis improvement were recorded after the consumption of Runjing extract.

It is important to emphasize that this study has limitations. Initially, the research was conducted for a short time period (60 days), which reflects the duration of spermatogenesis in male rats, without detecting the chronic side effects for an extended duration of exposure. Secondly, the histological examination was restricted to testicular investigation since the epididymal tissue was exhausted for spermiogram analysis.

## 5. Conclusions

To conclude, the present study suggests that the oral administration of aqueous herbal extracts, either individually or in combination, improves male fertility. Specifically, treatment with these extracts enhanced testosterone and LH levels, sperm count, antioxidant activity, and seminiferous tubule morphology. Additionally, the PI3K/AKT/mTOR signaling pathway and vimentin expression were upregulated, while PTEN expression was decreased. Among the individual plant extracts tested, OM was found to be the most effective in promoting fertility, while the mixture of plants proved to be the most efficient in enhancing fertility parameters. Therefore, our findings suggest that the plant mixture could be a promising natural alternative for male fertility enhancement. Future research should focus on investigating the effect of these plants on other signaling molecules and pathways involved in spermatogenesis, as well as ascertain their mode of action and optimal dosage in humans. Additionally, it is important to evaluate their possible toxicological effects on other organs in order to assure their safety if used for a long period of time.

## Figures and Tables

**Table 1:** Changes in body weight (g), testes weight, and gonadosomatic index among experimental groups.

Experimental Groups	Body Weight (g)	Testes Weight (g)	Gonadosomatic Index (%)
Ctrl	348.75 ± 17.95	2.50 ± 0.29 ^a^	0.71 ± 0.07 ^a^
LS	378.75 ± 12.64	3.05 ± 0.07 ^ab^	0.80 ± 0.03 ^ab^
OM	368.75 ± 12.14	3.33 ± 0.12 ^b^	0.90 ± 0.02 ^b^
FH	393.75 ±8.75	3.06 ± 0.12 ^ab^	0.77 ± 0.02 ^ab^
ES	397.50 ±19.84	3.29 ± 0.09 ^b^	0.82 ± 0.02 ^ab^
MIX	371.25 ± 11.43	3.10 ± 0.05 ^ab^	0.83 ± 0.03 ^ab^
VitE	408.75 ± 11.43	3.28 ± 0.09 ^b^	0.80 ± 0.01 ^ab^
Results of one-way ANOVA (groups)	F = 2.138	F = 3.990	F = 2.292
	*p* > 0.05	*p* < 0.05	*p* > 0.05

Data are presented as means ± SEM (n = 4/group). Means in the same column with different letters are significantly different. Values with *p* < 0.05 are significant, n = 4/group. Ctrl, normal control; LS, *Lepidium sativum* aqueous extract-treated; OM, *Origanum majorana* aqueous extract-treated; FH, *Ferula hermonis* aqueous extract-treated; ES, *Eruca sativa* aqueous extract-treated; MIX, plant mix-treated; VitE, vitamin E-treated.

**Table 2:** Changes in sperm parameters (concentration, motility, and viability) among the experimental groups.

Experimental Groups	Sperm Concentration (million/mL)	Sperm Motility (%)	Sperm Viability (%)	Mean Johnsen’s Score
Ctrl	16.75 ± 0.85 ^a^	31.75 ± 3.94	43.50 ± 1.55	9.30 ± 0.26
LS	35.50 ± 2.25 ^ab^	33.50 ± 2.36	41.75 ± 3.12	9.60 ± 0.30
OM	57.75 ± 12.18 ^b^	31.75 ± 1.44	45.00 ± 2.20	9.70 ± 0.21
FH	54.25 ± 6.14 ^b^	34.00 ± 2.68	40.00 ± 0.82	9.80 ± 0.13
ES	39.75 ± 3.77 ^ab^	36.00 ± 2.45	46.75 ± 2.36	9.60 ± 0.30
MIX	50.25 ± 1.65 ^b^	37.50 ± 2.79	46.50 ± 3.95	9.90 ± 0.10
VitE	41.25 ± 1.88 ^ab^	42.50 ± 2.50	50.25 ± 4.09	9.50 ± 0.40
Results of one-way ANOVA (groups)	F = 6.322	F = 1.554	F = 1.481	F = 0.559
	*p* < 0.001	*p* > 0.05	*p* > 0.05	*p* > 0.05

Data are presented as means ± SEM. Means in the same column with different letters are significantly different. Values with *p* < 0.05 are significant, n = 4/group. Ctrl, normal control; LS, *Lepidium sativum* aqueous extract-treated; OM, *Origanum majorana* aqueous extract-treated; FH, *Ferula hermonis* aqueous extract-treated; ES, *Eruca sativa* aqueous extract-treated; MIX, plant mix-treated; VitE, vitamin E-treated. Superscript letters (a and b) indicate significance within groups. The values marked with the same superscript letter are similar (*p* > 0.05), whereas those marked with different ones are significantly different (*p* < 0.05).

**Table 3:** Changes in antioxidant/oxidant parameters among the experimental groups.

Experimental Groups	N	Testes GSH (nmol/mg Protein)	Testes SOD (U/mg Protein)	Testes MDA (nmol/g Protein)
Ctrl	4	6.40 ± 1.04 ^a^	17.93 ± 2.583	21.41 ± 3.47
LS	4	8.78 ± 1.72 ^ab^	18.66 ± 0.955	28.89 ± 1.20
OM	4	16.03 ± 1.81 ^bd^	16.29 ± 0.902	21.03 ± 1.92
FH	4	8.13 ± 1.01 ^ae^	14.53 ± 0.622	24.34 ± 3.79
ES	4	14.61 ± 1.49 ^bef^	19.15 ± 1.727	19.16 ± 1.17
MIX	4	18.86 ± 1.78 ^cdf^	18.47 ± 0.489	22.78 ± 0.67
VitE	4	12.38 ± 2.51 ^adf^	20.59 ± 1.049	21.94 ± 4.13
Results of one-way ANOVA (groups)		F = 7.374	F = 2.102	F = 1.355
		*p* < 0.001	*p* > 0.05	*p* > 0.05

Data are presented as means ± SEM. Means in the same column with different letters are significantly different. Values with *p* < 0.05 are significant, n = 4/group. Ctrl, normal control; LS, *Lepidium sativum* aqueous extract-treated; OM, *Origanum majorana* aqueous extract-treated; FH, *Ferula hermonis* aqueous extract-treated; ES, *Eruca sativa* aqueous extract-treated; MIX, plant mix-treated; VitE, Vitamin E-treated. Superscript letters (a, b, c, d, e, and f) indicate significance within groups. The values marked with the same superscript letter are similar (*p* > 0.05), whereas those marked with different ones are significantly different (*p* < 0.05).

## Data Availability

Data are contained within the article and the Supplementary Materials.

## Supplementary Materials

The following supporting information can be downloaded at https://www.mdpi.com/article/10.3390/life14121620/s1: Figure S1: Representative chromatogram of the HPLC analysis performed on polyphenols standards: (a) gallic acid and catechin, (b) protocatechic acid, (c) hydroxybenzoic acid, (d) chlorogenic acid, (e) caffeic acid, (f) *p*-coumaric acid, (g) rutin, (h) ellagic acid, and (i) trans-cinnamic acid. Figure S2: Representative chromatogram of the HPLC analysis performed on the selected herbs (a) LS, *Lepidium sativum* aqueous extract (b) OM, *Origanum majorana* aqueous extract (c) FH, *Ferula hermonis* aqueous extract (d) ES, *Eruca sativa* aqueous extract and (e) MIX, plant mix. The chromatograms were obtained at different wavelengths for each extract for the detection of phenolic compounds.
