# Supplementary material for: Evaluation of Selected Folk Herbs on the Fertility of Sprague Dawley Male Rats: Biochemical, Histological, and Molecular Investigations
Source: Life (Basel). 2024 Dec 6;14(12):1620. doi: 10.3390/life14121620 (PMC11676069; doi:10.3390/life14121620)
Supplement: Supplementary file 1 [file life-14-01620-s001.zip › life-3329374-supplementary.pdf]

Supplementary material

(a)

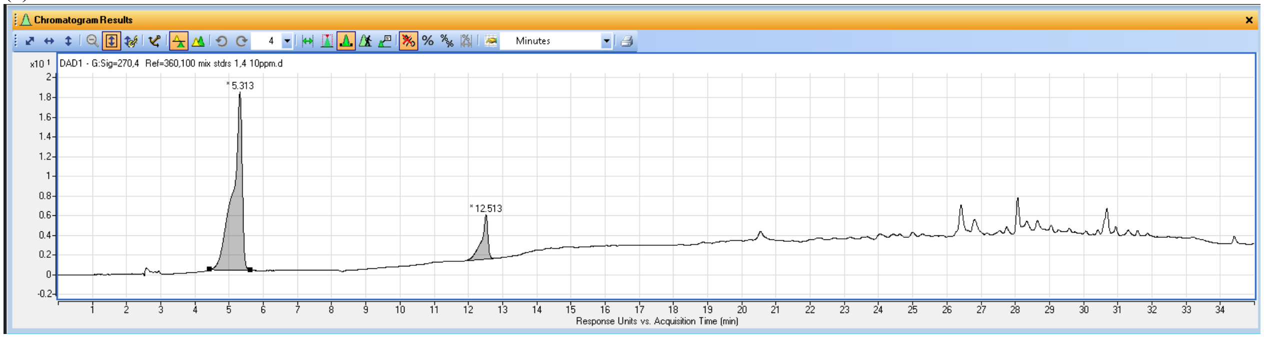

(b)

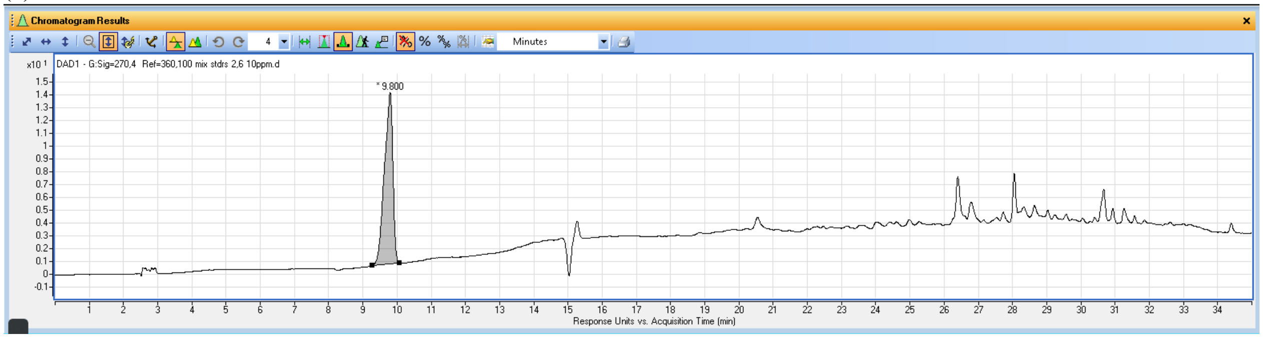

(c)

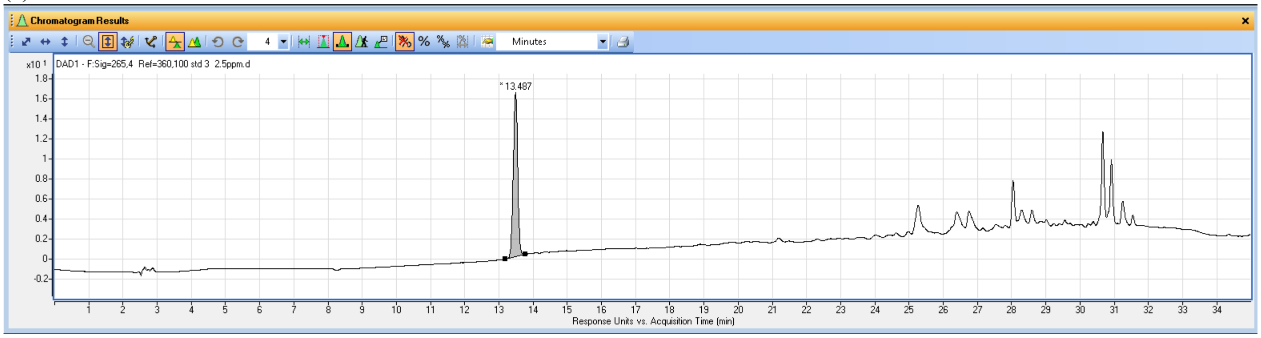

(d)

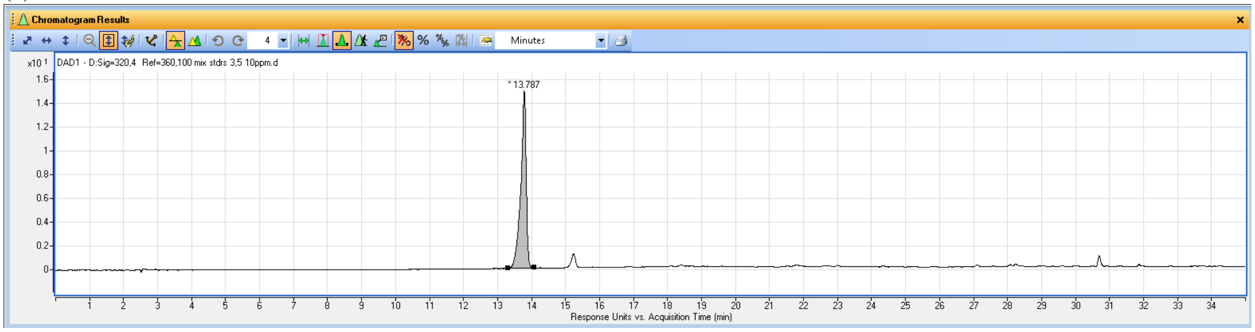

(e)

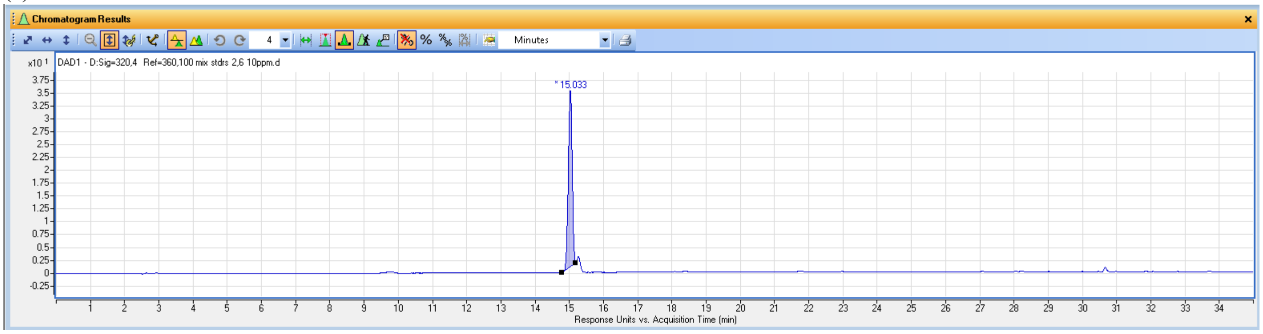
Chromatogram Results
Minutes
x10 1
DAD1 - D:Sig=320,4 Ref=360,100 mix stdrs 2,6 10ppm.d
* 15.033
Response Units vs. Acquisition Time (min)

(f)

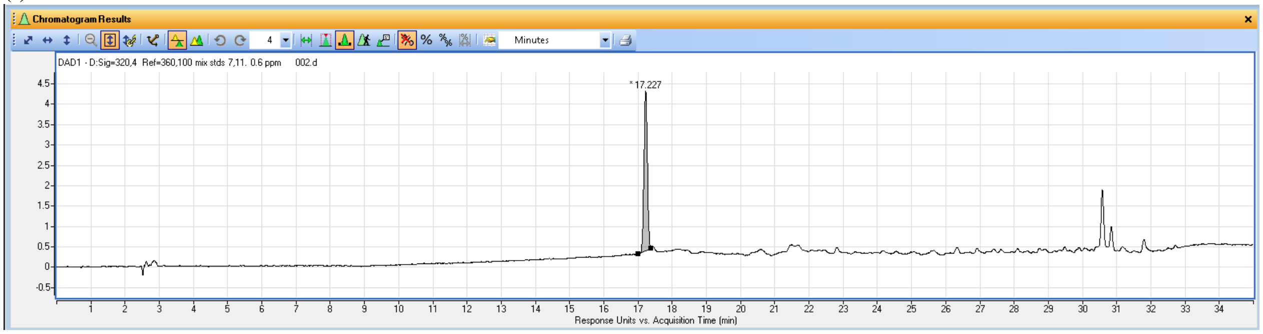
Chromatogram Results
Minutes
DAD1 - D:Sig=320,4 Ref=360,100 mix stds 7,11. 0.6 ppm 002.d
* 17.227
Response Units vs. Acquisition Time (min)

(g)

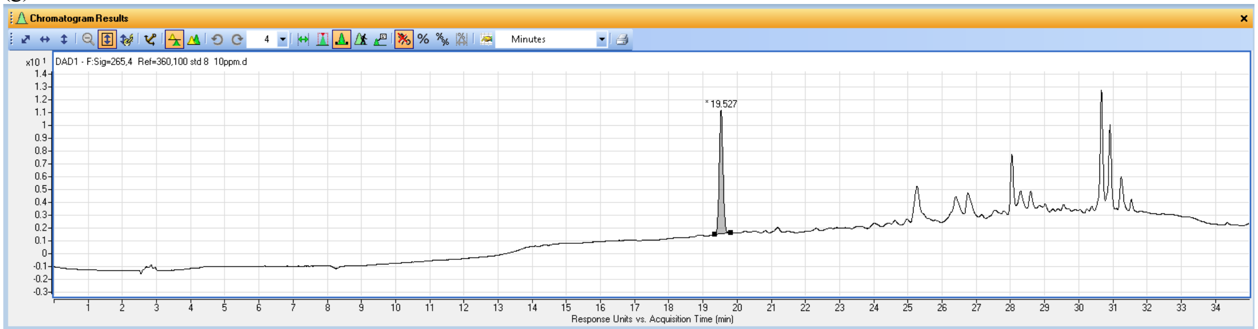
Chromatogram Results
Minutes
x10 1
DAD1 - F:Sig=265,4 Ref=360,100 std 8 10ppm.d
* 19.527
Response Units vs. Acquisition Time (min)

(h)

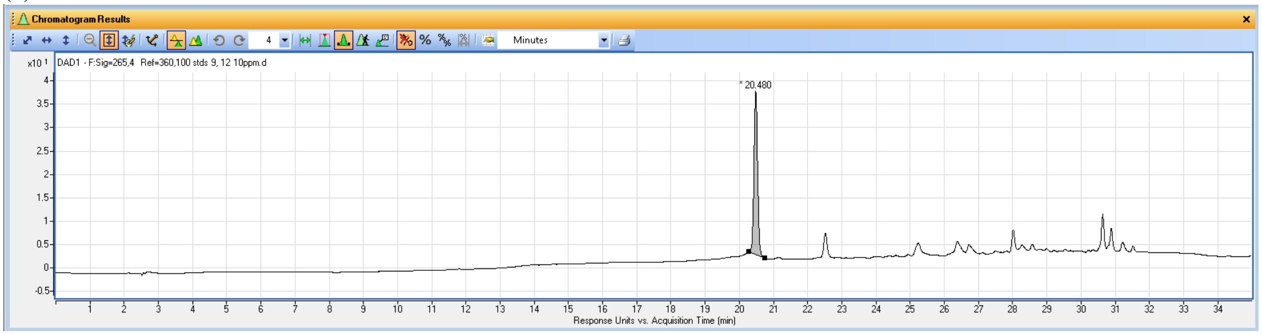
Chromatogram Results
Minutes
x10 1
DAD1 - F:Sig=265,4 Ref=360,100 stds 9, 12 10ppm.d
* 20.480
Response Units vs. Acquisition Time (min)

(i)

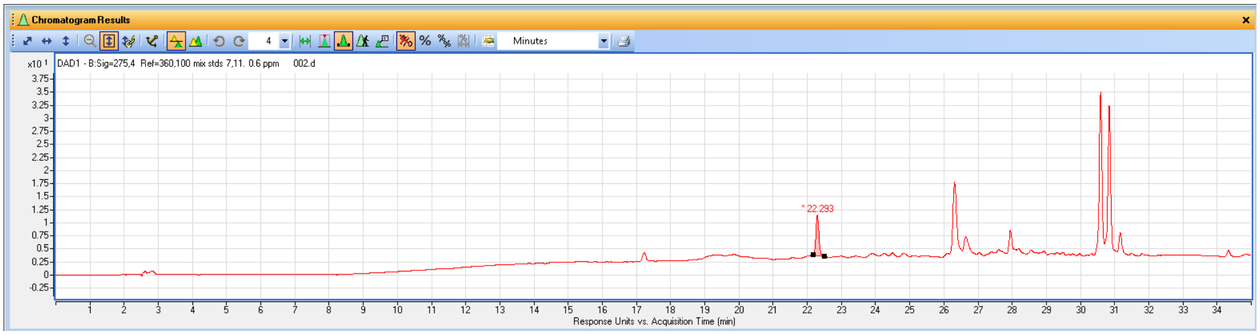

Figure S1: Representative chromatogram of the HPLC analysis performed on polyphenols standards: (a) gallic acid and catechin, (b) protocatechic acid, (c) hydroxybenzoic acid, (d) chlorogenic acid, (e) caffeic acid, (f) coumaric acid, (g) rutin, (h) ellagic acid, and (i) trans-cinnamic acid.

(a)

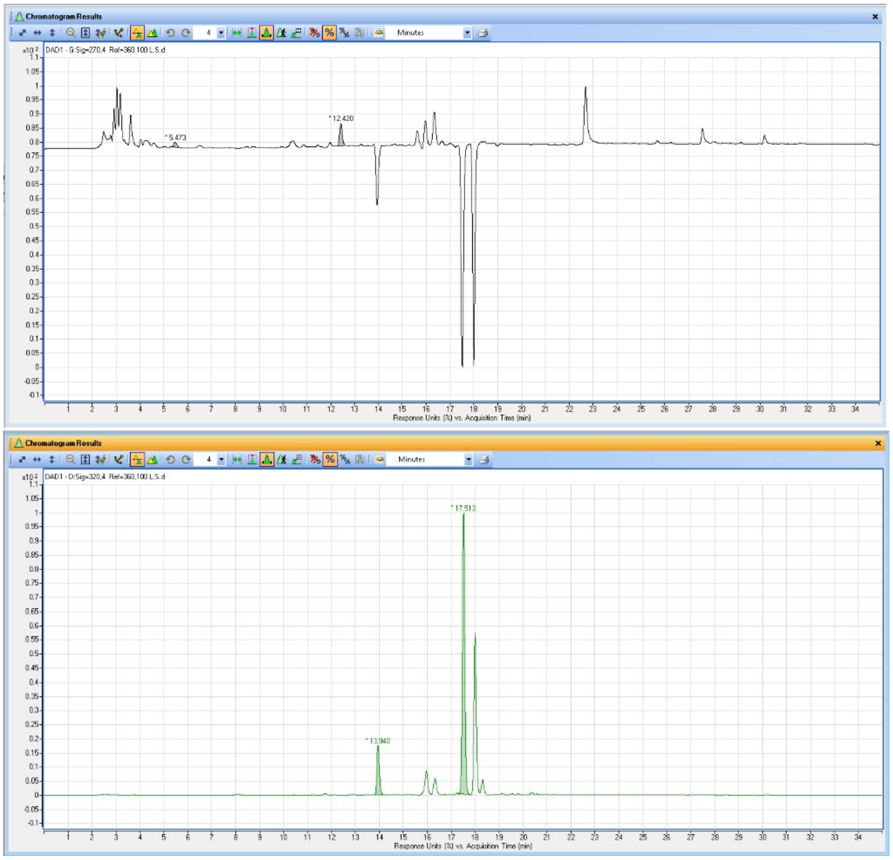

(b)

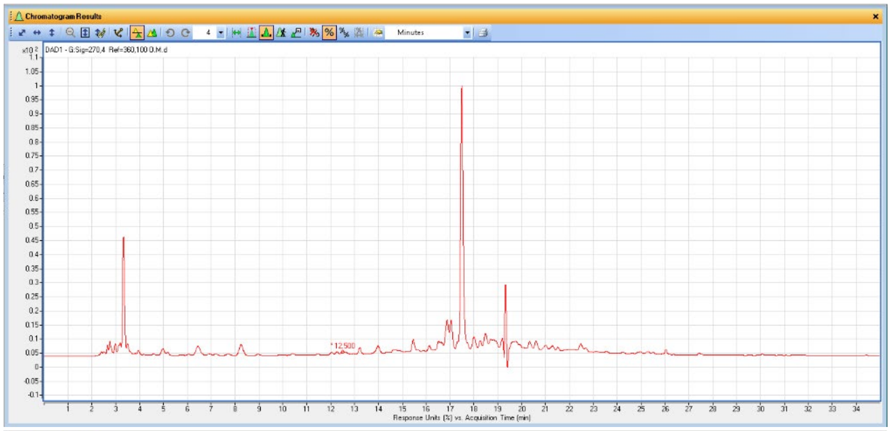
Chromatogram Results
Minutes
DAD1 - G:Sig=270,4 Ref=360,100 O.M.d
* 12,500
Response Units (%) vs. Acquisition Time (min)

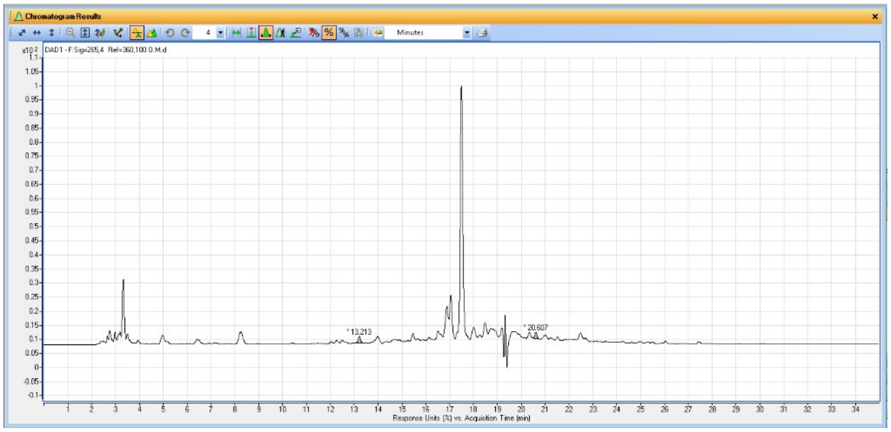
Chromatogram Results
Minutes
DAD1 - F:Sig=285,4 Ref=360,100 O.M.d
* 13,213
* 20,607
Response Units (%) vs. Acquisition Time (min)

(c)

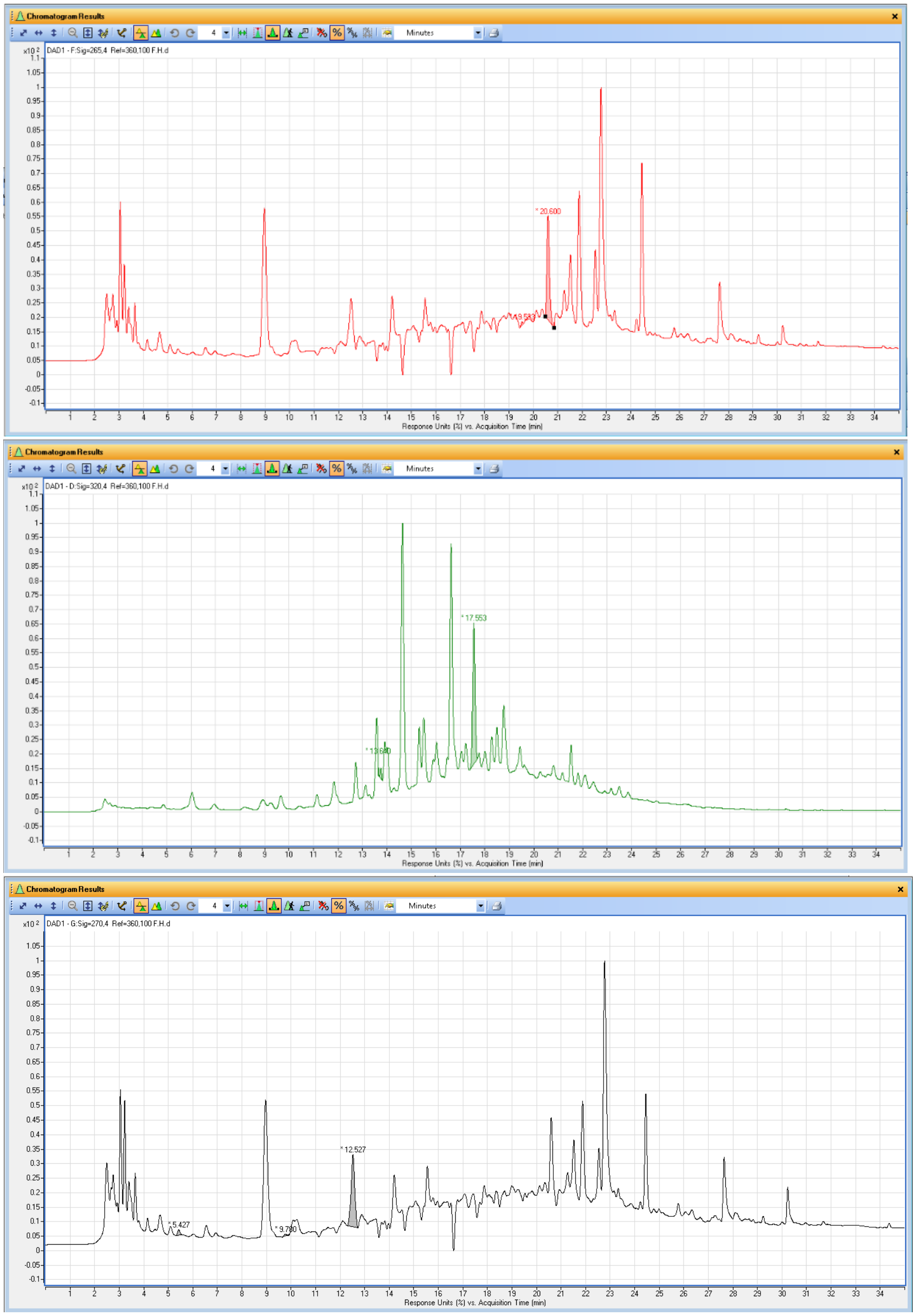
Chromatogram Results
Minutes
x10 2
DAD1 - F:Sig=265,4 Ref=360,100 F.H.d
* 20.600
Response Units (%) vs. Acquisition Time (min)
Chromatogram Results
Minutes
x10 2
DAD1 - D:Sig=320,4 Ref=360,100 F.H.d
* 17.553
Response Units (%) vs. Acquisition Time (min)
Chromatogram Results
Minutes
x10 2
DAD1 - G:Sig=270,4 Ref=360,100 F.H.d
* 12.527
* 5.427
* 9.780
Response Units (%) vs. Acquisition Time (min)

(d)

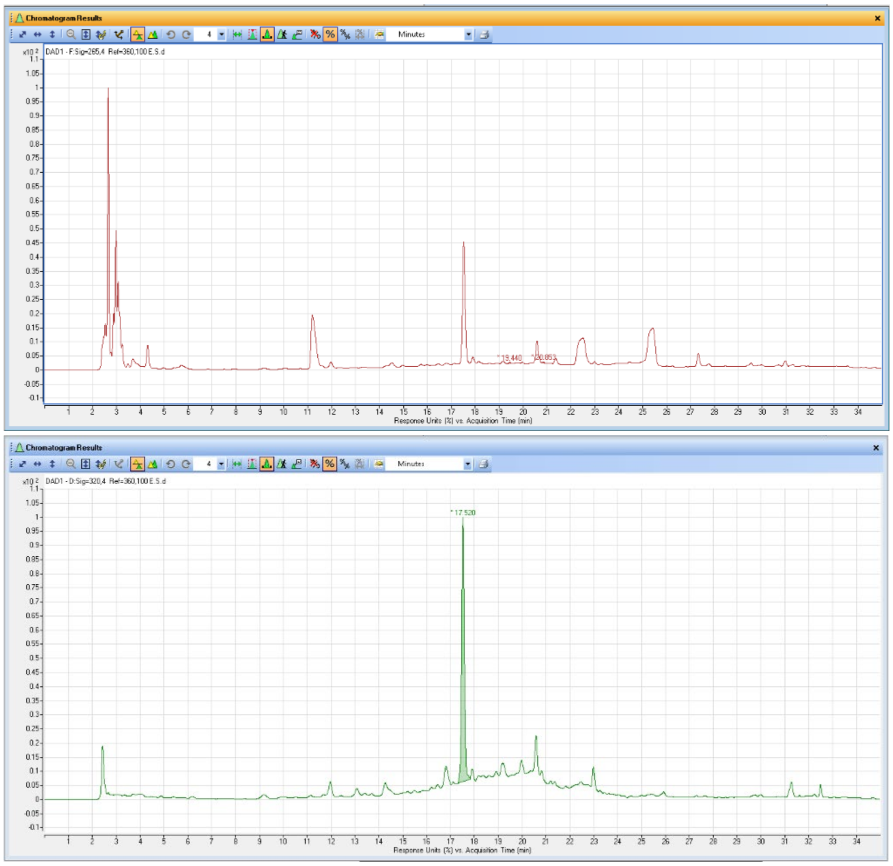
Chromatogram Results
DAD1 - F:Sig=265,4 Ref=360,100 E.S.d
Response Units (%) vs. Acquisition Time (min)
Chromatogram Results
DAD1 - D:Sig=320,4 Ref=360,100 E.S.d
17.520
Response Units (%) vs. Acquisition Time (min)

(e)

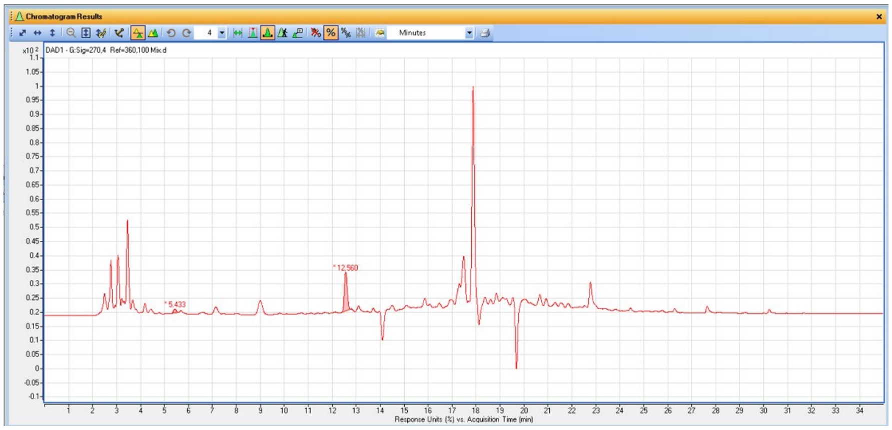
Chromatogram Results
DAD1 - G:Sig=270,4 Ref=360,100 Mix.d
5.433
12.560
Response Units (%) vs. Acquisition Time (min)

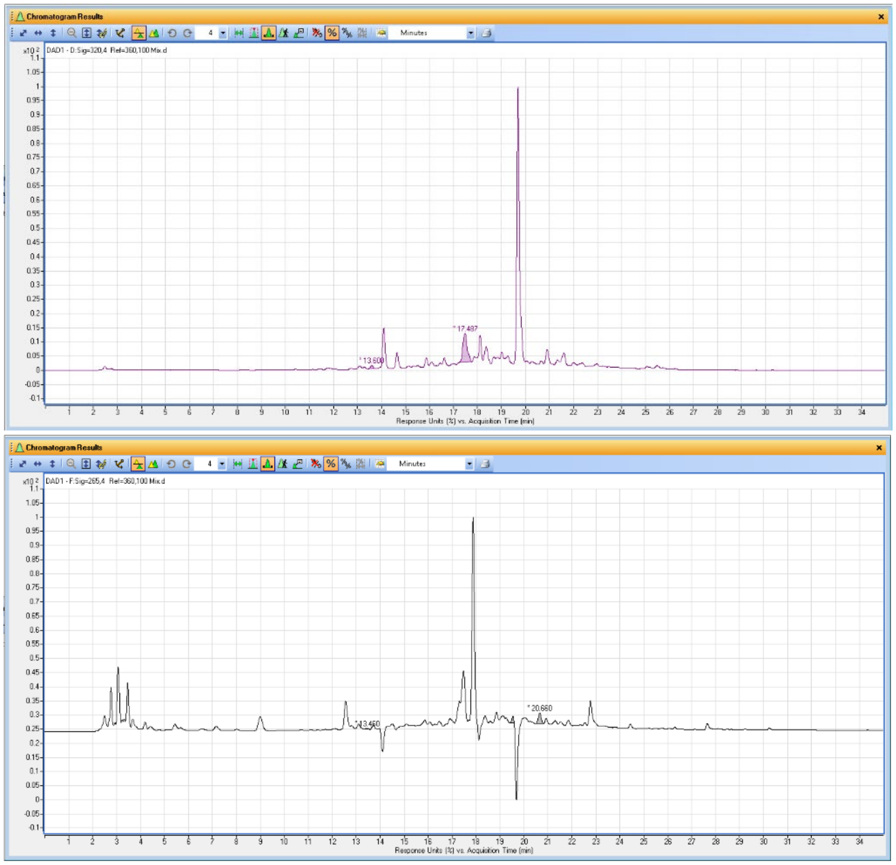

Figure S2. Representative chromatogram of the HPLC analysis performed on the selected herbs (a) LS, *Lepidium sativum* aqueous extract (b) OM, *Origanum majorana* aqueous extract (c) FH, *Ferula hermonis* aqueous extract (d) ES, *Eruca sativa* aqueous extract and (e) MIX, plant mix. The chromatograms were obtained at different wavelengths for each extract for the detection of phenolic compounds.
